# Preparation and characterization of a novel silicon-modified nanobubble

**DOI:** 10.1371/journal.pone.0178031

**Published:** 2017-05-30

**Authors:** Jia Liu, Bo Zhang, Maotong Li, Meijun Zhou, Fei Li, Xiuxian Huang, Min Pan, Li Xue, Fei Yan

**Affiliations:** 1 Department of Echocardiography, Fourth Affiliated Hospital of Harbin Medical University, Harbin, Heilongjiang, China; 2 Department of Echocardiography, Shanghai Eastern Hospital Affiliated to Tongji University, Shanghai, China; 3 CT Room, The First Affiliated Hospital of Harbin Medical University, Harbin, China; 4 Department of Ultrasonography, The Third Affiliated Hospital of Southern Medical University, Guangzhou, China; 5 Paul C. Lauterbur Research Center for Biomedical Imaging, Institute of biomedical and Health Engineering, Shenzhen Institutes of Advanced Technology, Chinese Academy of Sciences, Shenzhen, China; 6 Department of Echocardiography, Shenzhen Hospital of Guangzhou University of Chinese Medicine, Shenzhen, China; The Ohio State University, UNITED STATES

## Abstract

Nanobubbles (NBs) opened a new field of ultrasound imaging. There is still no practical method to control the diameter of bubbles. In this study, we developed a new method to control the size by incorporating of silicon hybrid lipids into the bubble membrane. The range of particle size of resulting NBs is between 523.02 ± 46.45 to 857.18 ± 82.90, smaller than the conventional microbubbles. The size of resulting NBs increased with the decrease in amount of silicon hybrid lipids, indicating the diameter of NBs can be regulated through modulating the ratio of silicon hybrid lipids in the bubble shell. Typical harmonic signals could be detected. The in vitro and in vivo ultrasound imaging experiments demonstrated these silicon-modified NBs had significantly improved ultrasound contrast enhancement abilities. Cytotoxicity assays revealed that these NBs had no obvious cytotoxicity to the 293 cell line at the tested bubble concentration. Our results showed that the novel NBs could use as nanoscale ultrasound contrast agents, providing the foundation for NBs in future applications including contrast-enhanced imaging and drug/gene delivery.

## Introduction

Noninvasive medical imaging has played more and more important roles in clinical practice. A good example of noninvasive imaging is ultrasound imaging, which allows for repeated, non-invasive, and direct monitoring of the processes of diseases [1, 2]. The emergence of ultrasound contrast agents (UCAs) is a milestone in the development of ultrasound imaging and offers a tremendous advantage for contrast enhanced ultrasound (CEUS) due to its high contrast and temporal resolution [3–5]. To date, commercial UCAs with diameters of 1–8 μm are typically designed to serve only as blood pool agents, but not for surrounding tissues or cells [6–8]. Therefore, a small particle size bubble is required for ultrasound contrast-enhanced agents.

Nanoscale bubbles (NBs), with particle sizes less than 1 μm, have shown great promise in ultrasound imaging [9]. Through the enhanced permeability and retention (EPR) effects, nanobubbles could be transferred from blood vessels into surrounding tissues and be imaged by ultrasound after accumulation [10]. Recently, nanoscale ultrasound contrast agents have been developed via a variety of methods including gradient separation by gravitational forces, physical filtration or floatation. All of them require the addition of amphiphilic surfactants during preparation, which would bring with some side-effects on cells [11, 12]. The fabrication of nanodroplets through encapsulating different perfluorocarbons such as perfluoropentane (PFP) or perfluorohexane (PFH) into nanoscale particles is an alternative approach to produce UCAs. But they need some extra conditions to activate these nanoscale nanodroplets to produce microscale UCAs [13, 14]. In addition, the ultrasound scatter efficiency is often low due to the nanoscale size of NBs. As a result, ultrasound imaging is difficult for them. Although various shell-forming materials (polymers or phospholipids) have been used to attempt to improve contrast enhancement effect of NBs [15–19], the progress is still in the initial stages [20, 21]. Moreover, there is no practical method to control the diameter of bubbles.

Here we report our recent study of a novel silicon-modified nanobubble (SNB) by incorporating of cerasome-forming lipids (N-[N-(3-triethoxysilyl)propylsuccinamoyl]dihexadecylamine, CFL) into the MB membrane. In this work, the morphology, in vitro and in vivo imaging enhancement ability of nanobubbles were investigated and compared with microbubbles (MBs). Our study has provided experimental evidence that these novel nanobubbles have possible applications for ultrasound contrast agents in the future.

## Materials and methods

### Materials

1,2-distearoyl-sn-glycero-3-phosphatidylcholine (DSPC), 1,2- di s tearoyl—sn-glycero-3-phosphoethanolamine-N-[methoxy(polyethyleneglycol)-2000](DSPE-PEG2000) were purchased from Avanti Polar Lipids (Alabaster, AL, USA). (Alabaster, AL, USA). N-[N-(3-triethoxysilyl)propylsuccinamoyl]dihexadecylamine (CFL) was friendly provided by Dai Zhifei’s lab (Beijing, China) [22, 23]. Cell Counting Kit-8 (CCK-8) was purchased from Dojindo (Tokyo, Japan). The U87 human glioma cell line was purchased from the American Type Culture Collection. Six- to eight-week-old female BALB/c mice (about 20 g each) were obtained from Guangdong Medical Experimental Animal Center (Guangzhou, China).

### Preparation of silicon-modified nanobubbles

The SNBs were prepared using the method previously described by our laboratory [24]. Briefly, DSPC: CFL: DSPE–PEG2000 in the molar ratio 45:45:10 (SNB 1), 60:30:10 (SNB 2) or 80:10:10 (SNB 3) were dissolved in chloroform. The solvent was removed, and the dried lipid film was kept under vacuum for 2 h. The dried phospholipid blends were hydrated in 5 ml of buffer consisting of 0.1 M Tris (pH 7.4): glycerol: propylene glycol (80:10:10 by volume), followed by sonication in a bath sonicator (ULTR Asonik 28 X) for 10 min at 65°C. After the solution was cooled to room temperature, the gas phase in the vial was exchanged with perfluoropropane (C_3_F_8_), followed by mechanical vibration for 45 s. Unincorporated lipids were removed by washing in PBS. The control MBs were prepared using the same method with a DSPC: DSPE–PEG2000 molar ratio of 90:10 [25].

### Characterization of SNBs

The SNBs and control MB suspension (50 μl, 1×10^7^ particles/ ml) was applied to a microscope slide to observe their morphology. A coverslip was used to cover the sample before examining SNBs and control MB morphology under a microscope (Olympus IX71, Japan). Size distribution and concentration of bubbles were determined with an optical particle counter (Accusizer 780; Particle Sizing Systems, Santa Barbara, CA, USA). Particle size was measured by using a Zetasizer NANO ZS system (Malvern, UK).

### Detection of nonlinear signals and bubble destruction

Nonlinear imaging experiments were collected using a home-built imaging set-up. A pair of focal plane transducers were used in the acoustic scattering measurements. One of them was used as the transmitter with a center frequency of 5MHz, and the other was the receiver with a center frequency of 5MHz. In the experiment, bubbles were excited at the driving frequency of 3 MHz. A programmable pulse generator and amplifier of a radiofrequency drove the transducers at particular frequencies with sinusoidal pulse trains of 1 ms. Connected to an oscilloscope, the ultrasound signals were collected, and then analyzed by MATLAB (Mathworks). To test the capability of bubble destruction, SNBs and control MB were added into the agar wells in the same concentration. The ultrasound images in contrast mode before and after collapse were obtained by using of a VisualSonics Vevo 2100 high-frequency ultrasound scanner, and then the MATLAB was used for the Image post-processing. The signal differences were obtained by subtracting the signals of post-disruption images from the signals of pre-disruption.

### In vitro ultrasound imaging

The agar gel was made according to the following method. In brief, 2% agar was heated to form homogeneous agar gel solution. Eppendorf tubes (500 μl) were inserted into the solution (with a depth of 5 mm) until solidification of agar gels. Then, these eppendorf tubes were removed, and wells were formed in the agar gel. The different SNBs and control MB were added into the agar wells. The acoustic signals were examined by an 18-MHz transducer at lateral view equipped on a high-resolution US imaging system (Vevo 2100; VisualSonics). All of the imaging parameters (contrast gain, 35 dB; depth, 20 mm, width 23 mm; transmit power, 10%; dynamic range, 35 dB and frequency, 18 MHz) were kept constant during all of the image capturing.

### In vivo ultrasound imaging

All animal experiments were carried out according to the relevant laws and institutional guidelines for the care and use of laboratory animals. Protocols were approved by the Committee on the Ethics of Animal Experiments of Shenzhen Institutes of Advanced Technology, Chinese Academy of Science. Ultrasound imaging was conducted as described previously [26]. Briefly, seven mice were kept anesthetized by continuous inhalation of 2% isoflurane in oxygen at 2 L /min on a heated imaging platform during scanning. Breathing and heart rate were monitored by built-in sensors. Then the lower abdomen and ultrasound coupling gel was applied to the skin of the mice at the liver site. Ultrasound imaging was performed using the VisualSonics Vevo 2100 high-frequency ultrasound scanner operating in nonlinear contrast mode. All imaging parameters (contrast gain, 35 dB; depth, 20 mm, width 23mm; transmit power, 60%; dynamic range, 35 dB and frequency, 18 MHz) were kept constant during all of the image capturing.

SNBs or control MB (5×10^7^ bubbles in 100 ml PBS) were administered through the tail vein of the mice in random order to minimize bias, and injections were separated by about 30 min to clear bubbles from the blood circulation. Pressing the bottom (Pre Trigger) before injecting the SNBs and control MBs to store cine loop data for a predefined number of image frames. During experiments, images were acquired continuously at a frame rate of 15 frames/ s for 120 s. And then image processing and quantification were performed using Vevo2100 built-in software for subsequent documentation and analysis. After that, mice were sacrificed by CO_2_ euthanasia.

### Cytotoxicity assay

The cytotoxicity was assessed by Cell Counting Kit-8 (CCK-8, Dojindo, Kumamoto, Japan). The 293 cells were seeded in 96-well plates at a density of 2,000 cells/well with 100 μl/well of high glucose DMEM containing 10% fetal bovine serum (FBS). After the cells were cultured for 24 h, the different SNBs and control MBs (10^8^ bubbles/ml) were added in the cell wells for additional 24 h and 48 h. After that, 10 μl of CCK-8 solution was added into each well, the cells were incubated for 2 h. The absorbance at 450 nm was measured by a microplate reader (Synergy 4, BioTek, USA).

### Statistical analysis

SPSS 16.0 software was used for this statistical analysis. All values are expressed as the mean ± the standard error of the means (S.E.M) of three samples acquired from three independent experiments. Statistical comparisons were performed using the Student’s t-test. The statistically significant differences were set at p< 0.05 and very significant at p< 0.01.

## Results

### Synthesis of silicon-modified nanobubbles

**Fig 1:**
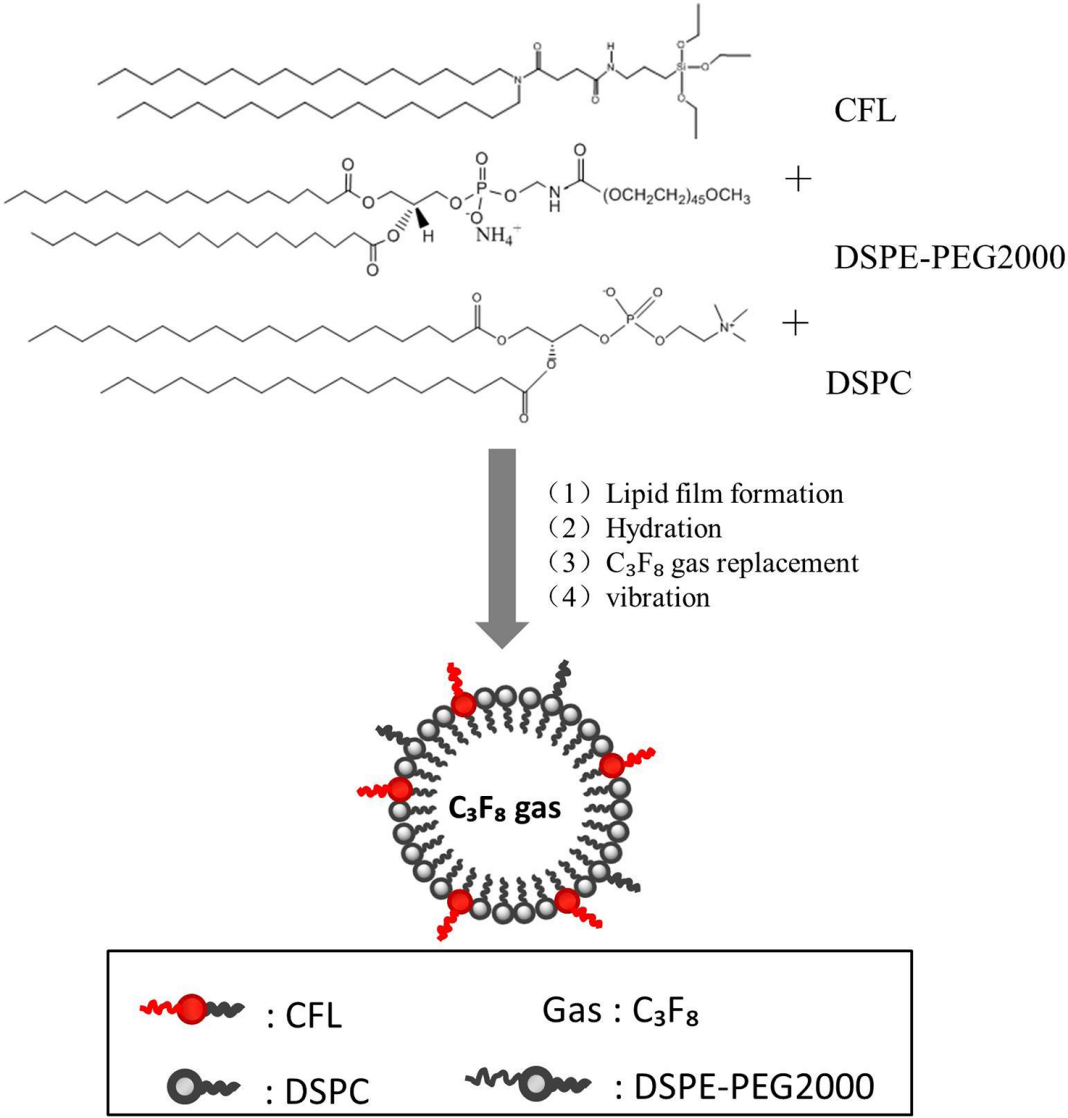
Schematic illustration of the procedure for fabrication of SNBs. Three formulations contained different amounts of CFL.

Fig 1 schematically illustrates the synthesis process of SNBs, including film formation, hydration, gas replacement and vibration. According to the method, three kinds of SNBs containing different amounts of CFL were obtained. SNB1 was prepared with formulation 1 (DSPC: DSPE-PEG2000: CFL = 45: 45: 10). SNB2 was obtained from formulation 2 (DSPC: DSPE-PEG2000: CFL = 60:30:10). SNB3 with formulation 3 (DSPC: DSPE-PEG2000: CFL = 80:10:10).

### Characterization of silicon-modified nanobubbles

**Fig 2:**
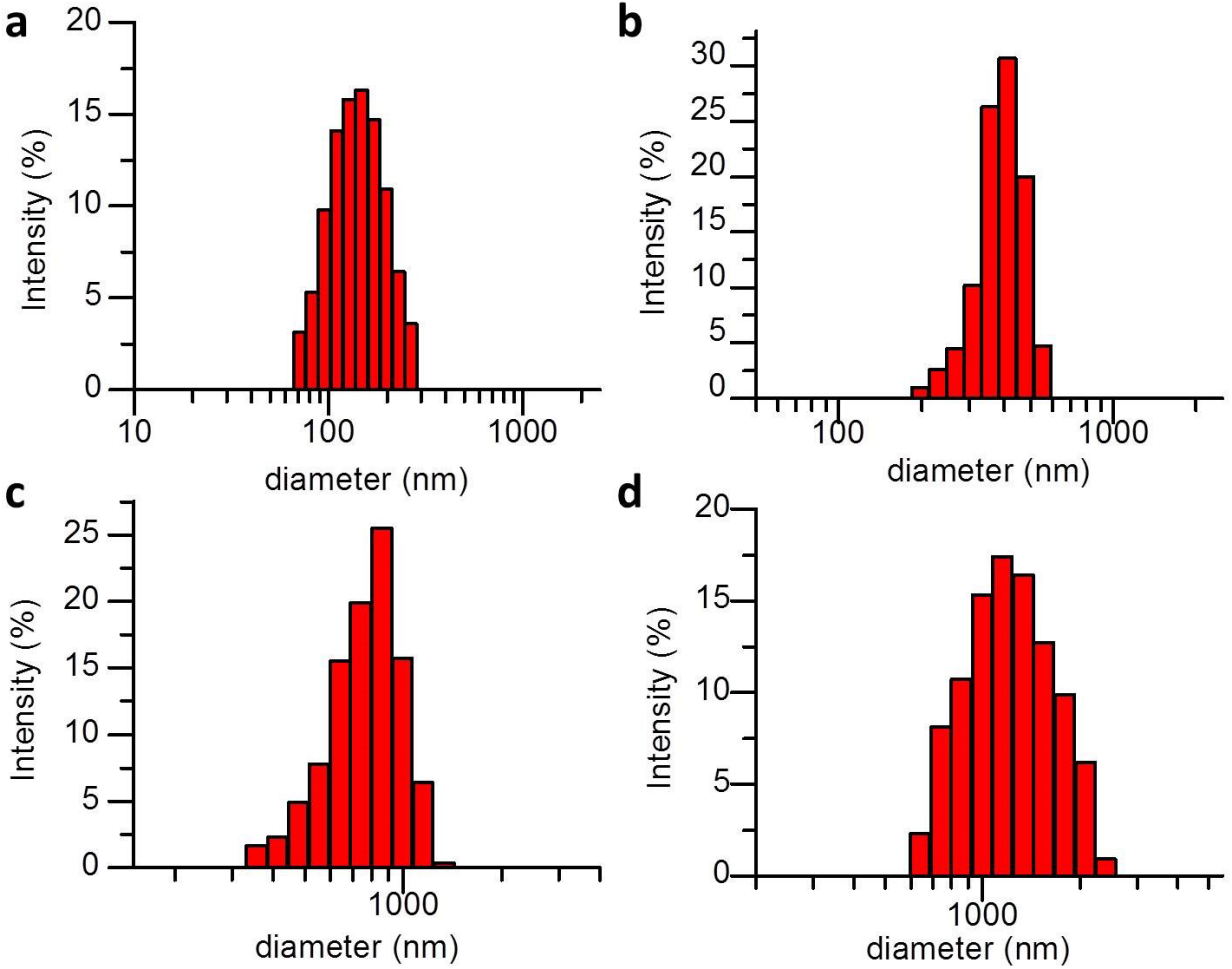
The size distribution of three different formulation SNBs and control MBs. (a) SNB 1, (b) SNB 2, (c) SNB 3 and (d) control MBs.

**Fig 3:**
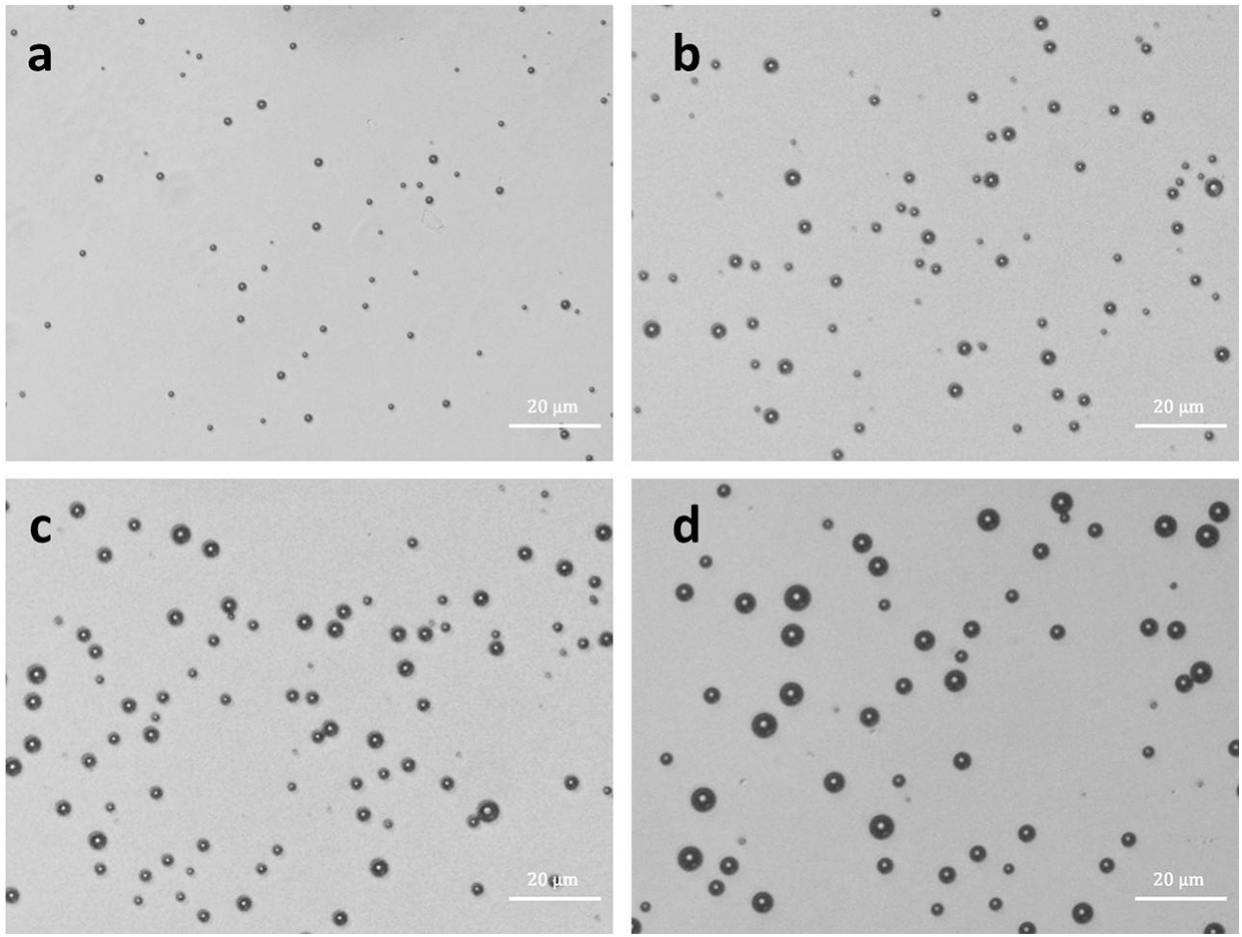
Bright-field images of (a) SNB 1, SNB 2 (b), SNB 3 (c) and control MBs (d), revealing spherical appearance and good dispersity (bar = 20 μm).

Typical size distributions of three kinds of SNB formulations (SNB 1, SNB 2 and SNB 3) and control MBs are illustrated in Fig 2, with 523.02 ± 46.45, 610.42 ± 53.06, 857.18 ± 82.90, 1317.34 ± 36.47 nm particle sizes in diameter, respectively. Interestingly, the size of resulting SNBs increased with the decrease of CFL in the lipid shell. Microscopic examination revealed that the resulting SNBs and control MB were spherical and had good dispersion (Fig 3).

### Detection of nonlinear signals of SNBs

**Fig 4:**
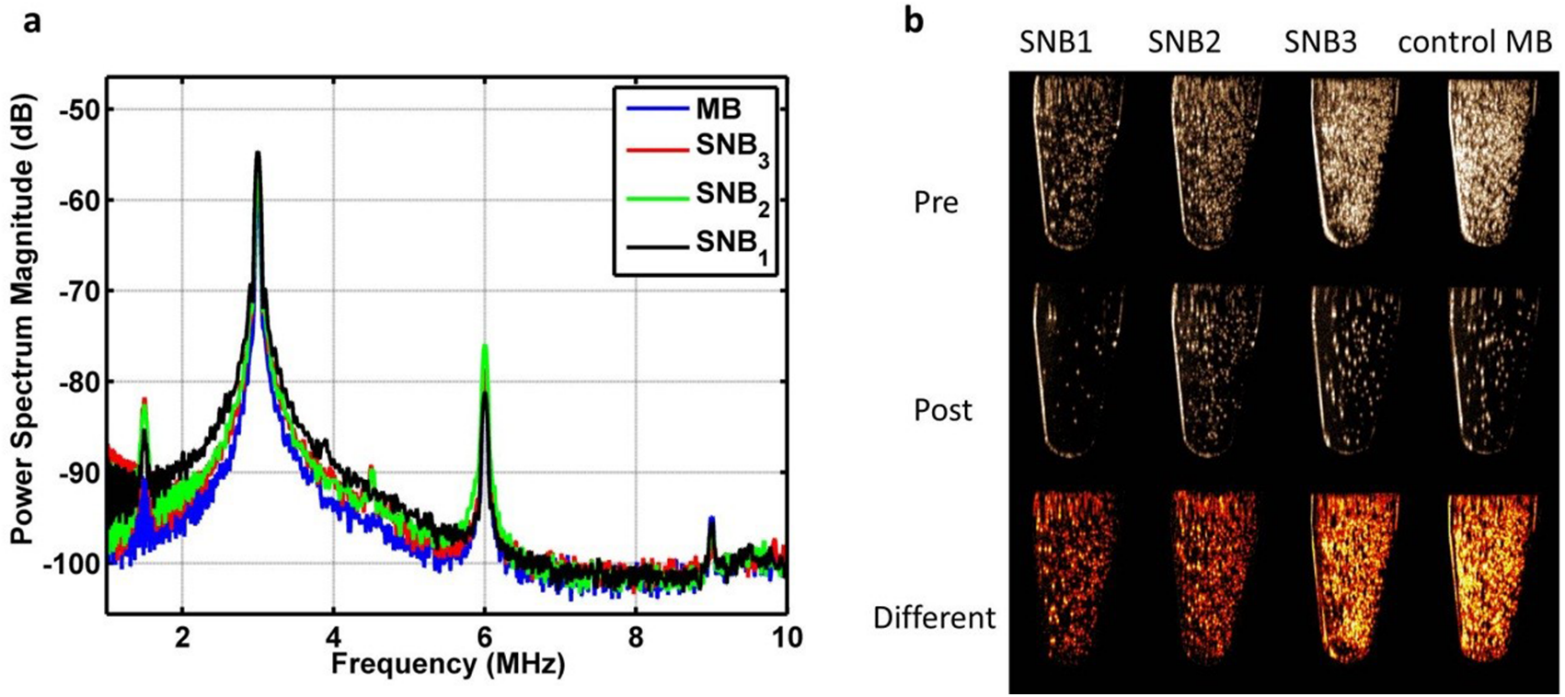
Nonlinear imaging and bubble destruction. (a) Power spectrum of signals backscattered from different SNBs and control MBs in response to 3 MHz transmitted pulses. Each point on the spectrum represents an average of 90 points from three samples (30 points per sample). (b) Ultrasound images of different SNBs and control MBs acquired before (Pre) and after (Post) destruction pulse ultrasound sequence, and the difference (Difference) between these images.

Harmonic backscatter is supposed to arise from oscillations of bubbles which responds incident pressure waves [27, 28]. Accordingly, the characterizations of the frequency response of bubbles to 3 MHz pulses over a receiving bandwidth of 1–10 MHz showed a distinct peak at the half, second and three harmonic frequency of 1.5, 6, 9 MHz, except that the fundamental peak at the transmitted frequency (Fig 4A). To further explore the capability of bubble destruction, we collapsed these bubbles through a disruption sequence. Significant signal decreases for three SNBs could be observed (Fig 4B), indicating they had comparable bubble destruction capability to MBs.

### In vitro ultrasound imaging

**Fig 5:**
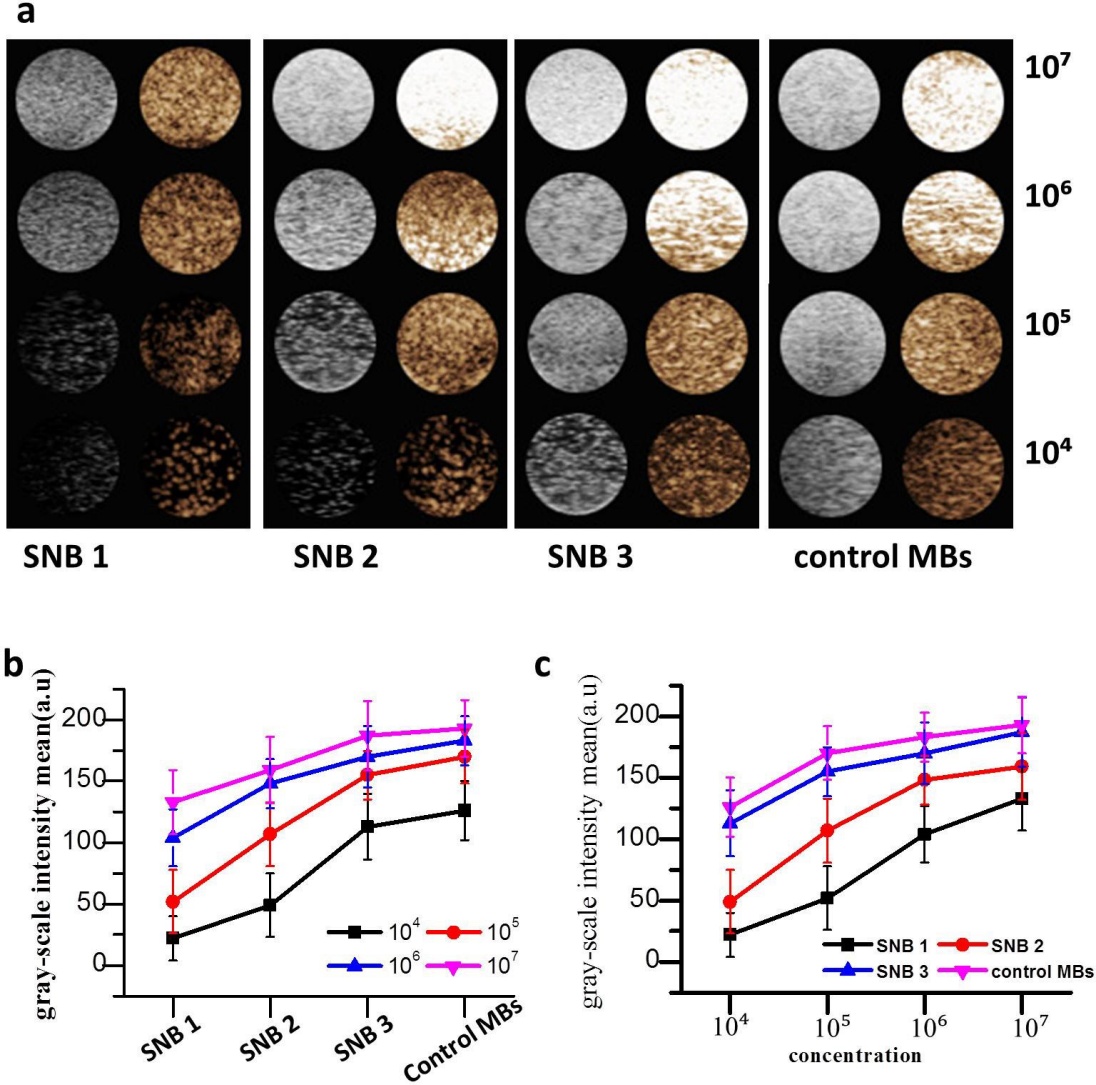
In vitro ultrasound imaging. (a) Ultrasound imaging of three different SNBs and control MBs in vitro. 1×10^4^, 1×10^5^, 1×10^6^ and 1×10^7^ on the right side of the figure mean the bubble concentrations. (b) Quantitative analysis of the B-mode signal intensities of SNBs and control MBs at different concentrations.

The in vitro ultrasound enhancement ability of the prepared SNBs were compared with that of control MBs. Fig 5A demonstrated the SNBs had good enhancement ability for ultrasound imaging. The less the CFL was used in SNBs, the stronger the ultrasound signals in the same bubble concentrations would be (Fig 5B). Also, for the same SNBs, the greater concentrations of bubbles were used, the stronger ultrasound signals would be obtained (Fig 5C). No significant difference was observed between SNB 3 and control MBs (p > 0.05), the gray-scale intensity of the SNB 3 was 190.600 ± 1.341 a.u (n = 5), while that of control MBs was 193.000 ± 1.224 a.u (n = 5).

### In vivo ultrasound imaging

**Fig 6:**
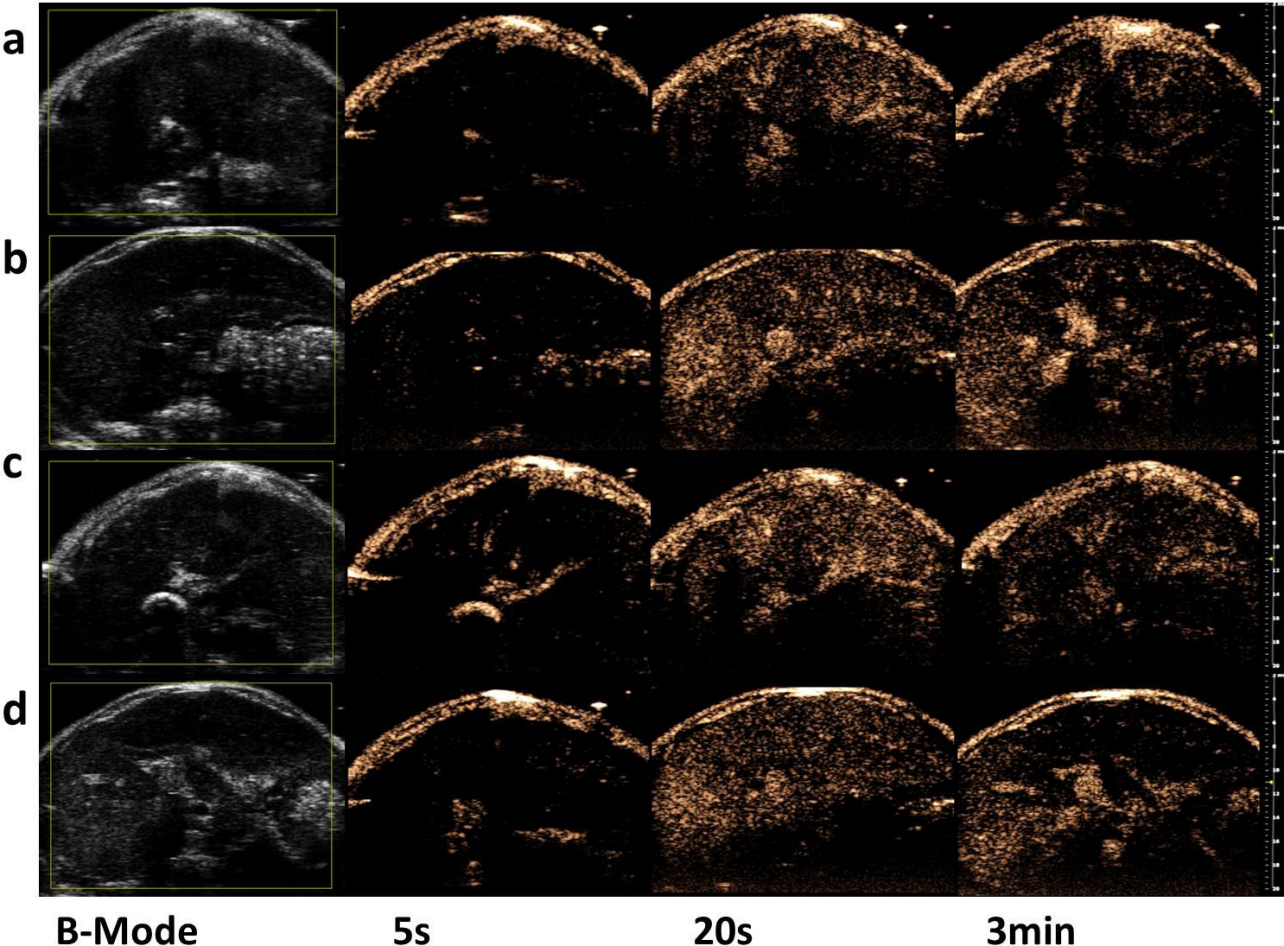
In vivo ultrasound image enhancement. Ultrasound-enhanced images of liver before and after vein injection of different SNBs and control MBs at 5 s, 20 s and 3 min. (a) SNB 1, (b) SNB 2, (c) SNB 3 and (d) control MBs.

To evaluate the in vivo imaging enhancement ability of the SNBs, liver imaging was performed on seven mice. No animals died during the experiments. After the injection of the different SNBs and control MBs into rat, the ultrasound signal intensity of SNBs in liver increased over time. Five seconds after the start of the injection, robust scattering signals could be observed in the inferior vena cava in nonlinear contrast images. As time passed, the ultrasound signals remained for 3 min and slowly decreased until no obvious ultrasound imaging signals could be seen at 5 min. Stronger ultrasound signals could be obtained when the less CFL was integrated into SNBs (Fig 6).

**Fig 7:**
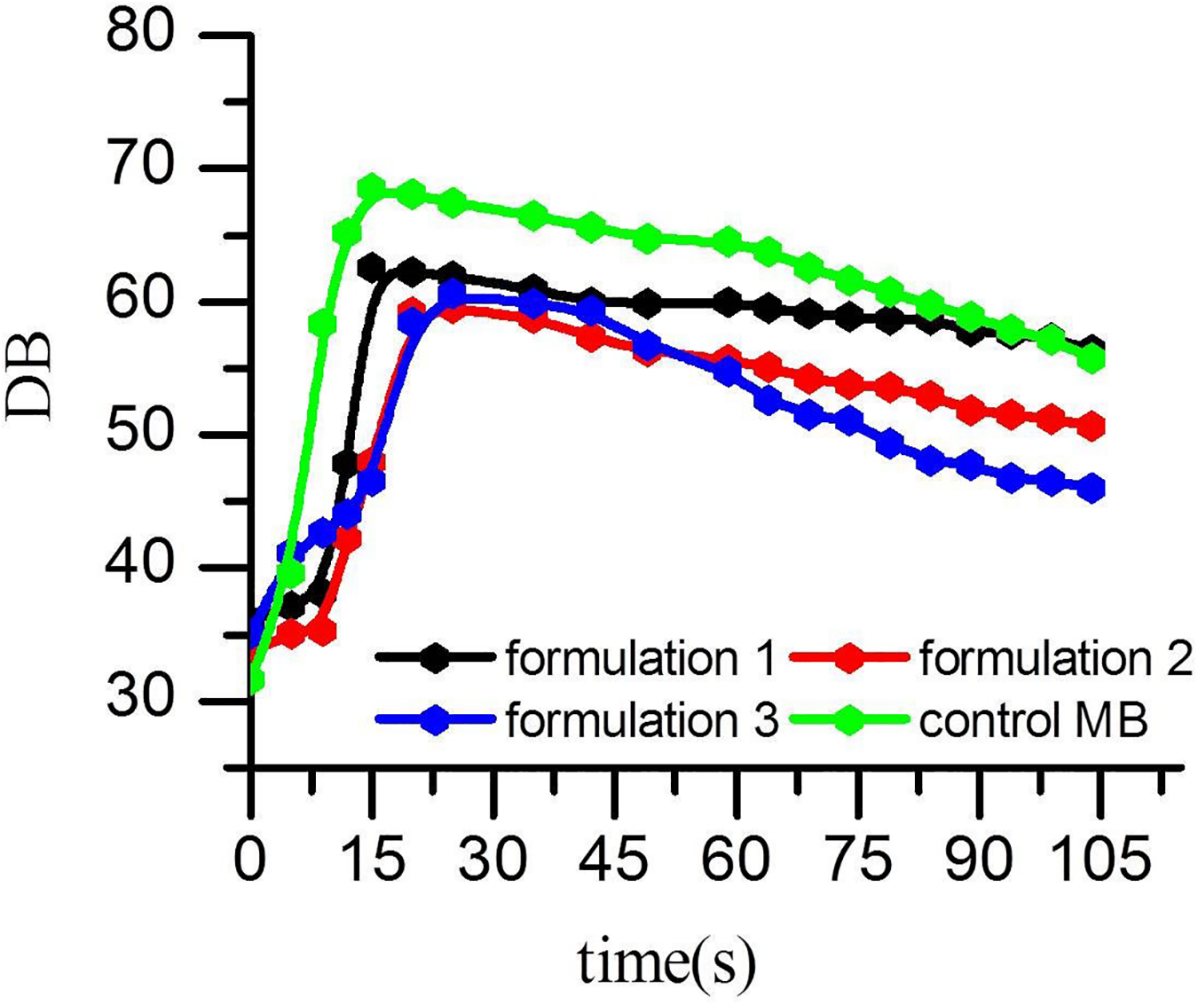
Quantitative analysis of the contrast-enhanced intensity in vivo after vein injection with different SNBs and control MBs.

Quantitative analysis of the contrast-enhanced sonograms revealed that the mean signal intensity for the liver injected with SNBs and control MBs (Fig 7 and Table 1). All of these results demonstrated that the three SNBs exhibited acceptable in vivo performance in ultrasound imaging. The time to peak intensity (TPI) for both the SNBs and control MBs was 15s. But the SNB1 showed the strongest ability of stability. It is in accordance with the results of in vitro experiments mentioned above.

**Table 1:** Comparison of ultrasound enhancement intensity (dB) by prepared SNBs and control MBs in vivo.

Time	0s	15s	30s	60s	120s
**SNB1**	36.01±4.9	62.53±4.6	60.96±3.4	59.97±5.9	56.35±2.6
**SNB2**	33.75±5.2	47.95±5.4	58.74±8.6	55.68±4.1	50.71±6.7
**SNB3**	35.10±6.3	46.53±6.2	59.88±5.7	54.78±4.2	45.98±3.9
**MB**	31.64±3.4	68.55±6.8	66.44±3.2	64.48±8.7	55.77±5.5

Data are presented as means ± standard deviation.

### Cytotoxicity assays

**Fig 8:**
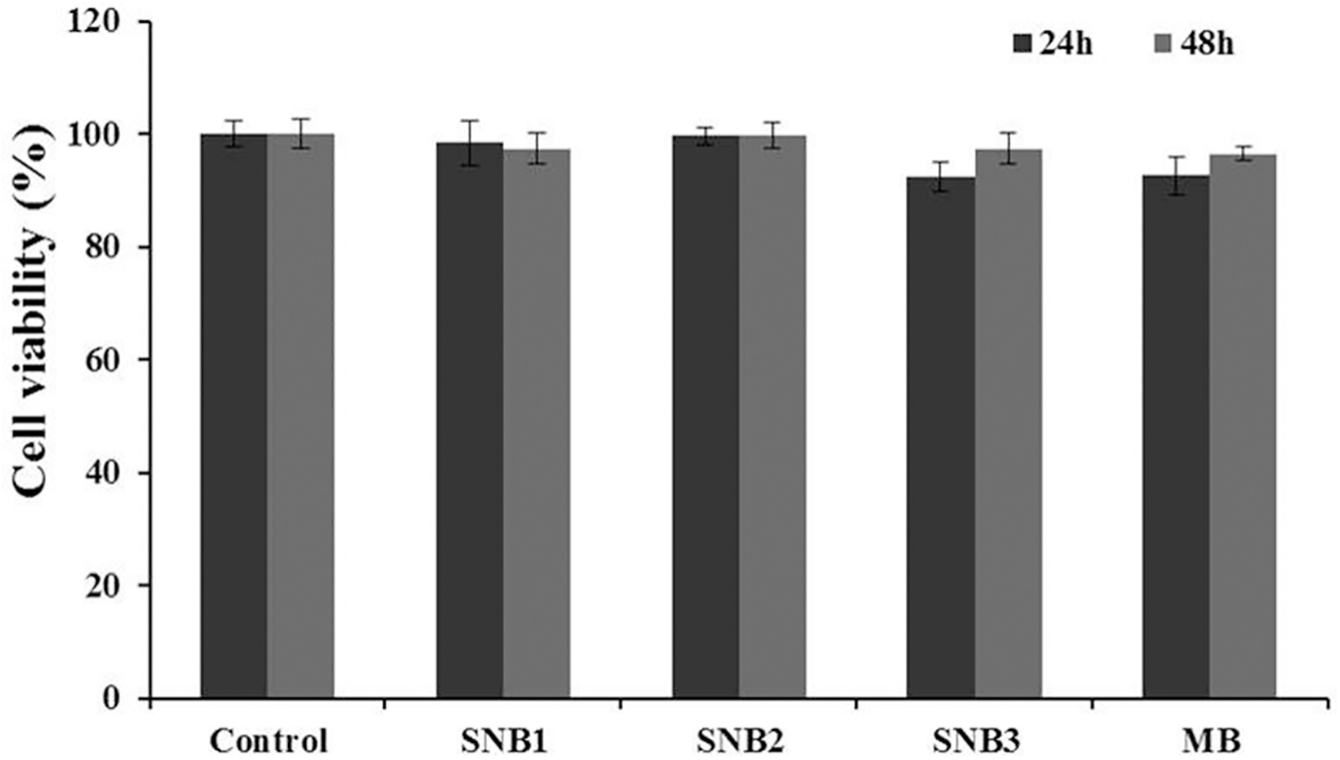
CCK-8 assay for 293 cells after 24 h and 48 h of incubation with different SNBs and control MBs in the same concentration (10^8^ /ml). n = 6.

The cytotoxicity of SNBs was evaluated using the cck-8 assay. The result was presented in Fig 8. The results revealed that the SNBs had no obvious cytotoxicity to the 293 cell line at the tested bubble concentration (p >0.05).

## Discussion

The particles size of UCAs is an increasingly important consideration in ultrasound imaging. Due to their small size, nanobubbles has greater potential in targeted molecular imaging and therapeutic application than that of microsized UCAs [17, 29, 30]. For example, NBs can efficiently penetrate through submucosal layers for tumor imaging [31, 32] and have longer circulation times in vivo [18, 19, 33]. The goal of this work was to investigate the characteristics of the novel silicon-modified nanobubbles as ultrasound contrast agents for the ultrasound imaging in vivo. Previous studies have suggested that there are lots of methods to form nanobubbles; such as controlling thickness of the phospholipid film [29]; differential centrifugation [11, 19, 30]. But the effect is not obvious, and there is no effective method to control the diameter of nanobubbles. In this study, we found a novel method to control the size of bubbles or nanobubbles by adding different amount of CFL.

The results from microscopy and optical particle counter provided direct observations of the nanobubble morphology and size. Microscopic image analysis at high magnification revealed that the resulting SNBs and control MBs were spherical and had good dispersion. The nanobubbles of SNB 1 did not appear to be spherical as previously reported [30, 31], because the SNBs were too small to distinguish between boundary.

The nanobubbles of SNB 3 had a good in vitro ultrasound image ability which was comparative with control MBs. The echogenicity of nanobubbles was mainly due to its inner gas, which allowed high scattering to ultrasound wave, as has been reported for other lipid-UCAs [32, 33]. In this work we found that the image enhancement ability is stronger with the increase of diameter. The main reason may contribute to the relative large diameter which can more effectively produce acoustic reflection. So the image enhancement ability of SNB 3 is better than that of SNB 1 at the same concentration.

In the in vivo ultrasound imaging, significantly enhanced ultrasound signals could be observed in the animal livers. Compared with the control MBs, SNBs showed better stability according to the quantitative analysis of the contrast-enhanced intensity in vivo. With the increase of CFL, the stability of SNBs would increase. This proved that silicon-modified nanoscale bubbles have contrast-enhanced ultrasound imaging ability [34–36] and are more stable than traditional MBs.

In summary, the approach of modulating the amount of CFL to regulate the diameter of nanobubbles is feasible for the preparation of nanobubbles with different diameters. The physical characteristics guaranteed a good image-enhanced ability of nanobubbles in vitro and in vivo. In the past years, microscale UCAs played an important role for highly vascularized organs or tumors in intra-vascular ultrasound imaging. The development of nanoscale UCAs might have better performance for poorly vascularized tumors. Our study in nanobubbles may be a step toward a new strategy to develop novel nanobubbles for ultrasound imaging and drug delivery.

## Conclusions

In this work, we have successfully developed a novel biocompatible nanoscale ultrasound contrast agent, and we can regulate the diameter of nanobubbles through modulating the ratio of CFL in the bubble shell. We demonstrated the diameter by microscope and found that the size of resulting bubbles increased with the decrease in amount of CFL. Also, we evaluated its echogenic ability both in vitro and in vivo. Our results showed that the novel SNBs could use as ultrasound contrast agents, providing the foundation for SNBs in future applications including contrast-enhanced imaging and drug/gene delivery.
